# Investigating the Multi-Target Pharmacological Mechanism of *Hedyotis diffusa* Willd Acting on Prostate Cancer: A Network Pharmacology Approach

**DOI:** 10.3390/biom9100591

**Published:** 2019-10-09

**Authors:** Yanan Song, Haiyan Wang, Yajing Pan, Tonghua Liu

**Affiliations:** 1 School of Traditional Chinese Medicine, Beijing University of Chinese Medicine, Beijing 100029, China; yanan.song@childrens.harvard.edu (Y.S.); lanyatou1986@126.com (H.W.); panyajing152@sina.com (Y.P.); 2 Newborn Medicine, Boston Children’s Hospital, Boston, MA 02115, USA

**Keywords:** *Hedyotis diffusa* Willd, prostate cancer, network pharmacology, natural products, herb

## Abstract

*Hedyotis diffusa* Willd (HDW) is one of the most well-known herbs used in the treatment of prostate cancer. However, the potential mechanisms of its anti-tumor effects have not been fully explored. Here, we applied a network pharmacology approach to explore the potential mechanisms of HDW against prostate cancer (PCa). We obtained 14 active compounds from HDW and 295 potential PCa related targets in total to construct a network, which indicated that quercetin and ursolic acid served as the main ingredients in HDW. Mitogen-activated Protein Kinase 8 (MAPK8), Interleukin 6 (IL6), Vascular Endothelial Growth Factor A (VEGFA), Signal Transducer and Activator of Transcription 3 (STAT3), Jun Proto-Oncogene (JUN), C-X-C Motif Chemokine Ligand 8 (CXCL8), Interleukin-1 Beta (IL1B), Matrix Metalloproteinase-9 (MMP9), C-C Motif Chemokine Ligand 2 (CCL2), RELA Proto-Oncogene (RELA), and CAMP Responsive Element Binding Protein 1 (CREB1) were identified as key targets of HDW in the treatment of PCa. The protein–protein interaction (PPI) cluster demonstrated that CREB1 was the seed in this cluster, indicating that CREB1 plays an important role in connecting other nodes in the PPI network. This enrichment demonstrated that HDW was highly related to translesion synthesis, unfolded protein binding, regulation of mitotic recombination, phosphatidylinositol and its kinase-mediated signaling, nucleotide excision repair, regulation of DNA recombination, and DNA topological change. The enrichment results also showed that the underlying mechanism of HDW against PCa may be due to its coordinated regulation of several cancer-related pathways, such as angiogenesis, cell differentiation, migration, apoptosis, invasion, and proliferation.

## 1. Introduction

Prostate cancer (PCa) is the second most common cancer in men, estimated to account for ~14.8% of total cancer diagnoses in men and the fifth leading cause of cancer-associated mortality [1,2,3,4]. According to current research, PCa is caused by the uncontrolled replication of abnormal cells in the prostate gland [2]. Although the cause of prostate cancer is not yet fully understood, many studies have suggested that several risk factors are associated with the development of prostate cancer, including environmental factors, family history, age, and lifestyle [5,6]. At present, various treatments are accessible for patients with PCa using Western medicine, including radical prostatectomy (RP, removal of the prostate), radiotherapy (use of high-energy X-rays to kill cancer cells), chemotherapy (use of chemicals to kill cancer cells), androgen deprivation therapy, and immune therapy [7,8,9,10,11,12,13]. However, these therapies are costly and often cause a series of adverse side effects, such as decreased libido, erectile dysfunction, hot flashes, night sweats, castration syndrome, including such symptoms as anemia, metabolic abnormalities, and radioactive inflammation, immune suppression, and others, all of which seriously affect the patient’s quality of life [14,15,16]. 

*Hedyotis diffusa* Willd (HDW, also known as *Hedyotis diffusa* herba and Oldenlandia diffusa, 白花蛇舌草, family Rubiaceae), is a traditional Chinese herb medicine historically used for thousands of years; it was traditionally widely used in heat-clearing, detoxification, and removal of blood stasis [17,18]. According to the literature, it has been used as a major component in several Chinese medicine formulas to treat cancer, as well as to provide a benefit against the adverse reactions of chemotherapy [19,20]. Moreover, its usage as an anti-tumor herb to treat different types of cancer, including prostate cancer, gastric cancer, colorectal cancer, liver cancer, breast cancer, and ovarian cancer, has been approved by research [21,22,23,24]. According to current pharmacology research, some ingredients with anti-cancer activities, such as anthraquinones, polysaccharides, flavones, hemiterpenes, polyphenols, and organic acids are contained in HDW [19,22,25]. It was also reported to possess a variety of bioactivities, such as anti-cancerous, anti-oxidant, anti-inflammatory, and hepatic-protective activities [17,23,24]. However, although many cancer studies confirmed that HDW exhibited noticeable anti-tumor effects, the potential mechanisms of its anti-tumor effects have not yet been fully explored [26,27]. 

It is widely known that herbal medicines (natural products) include “multi-component, multi-target, and multi-pathway” characteristics [26,27,28]. Traditional Chinese medicine (TCM) network pharmacology is a systematic method first proposed by Shao Li [29,30] based on the interaction network of herbs, compounds, targets, diseases, and genes [31]. This approach emphasizes the integration of bio-informatics, systems biology, and pharmacology, which not only interprets the complicated interactions between herbs and diseases at a systematic level, but also conforms to the systematic and holistic perspective of the TCM theory [32,33]. Therefore, in this study, we applied a network pharmacology approach to explore the pharmacological mechanisms of HDW as a therapy for PCa. Firstly, we screened for active compounds of HDW by evaluating their oral bio-availability (OB) and drug-likeness (DL) [34], and then the targets of the active ingredients in HWD were obtained. We screened the potential target genes of PCa via three databases (DisGeNET, OMIM, and Genecards) and then constructed the network by analyzing the potential interactions between the various target nodes. In addition, protein–protein interaction (PPI) data were obtained from the STRING database, and enrichment analyses (gene ontology (GO) and Kyoto Encyclopedia of Genes and Genomes (KEGG)) were performed to find the potential mechanism of HDW against PCa. To summarize, this study aimed to identify the potential targets and pathways of HDW as a therapy against PCa using the network pharmacology approach, and systematically elucidate the mechanism of HDW in the treatment of PCa.

## 2. Materials and Methods

### 2.1. Data Preparation

#### 2.1.1. Active Ingredients and Targets in HWD

The ingredients in HWD were obtained from the Traditional Chinese Medicine Systems Pharmacology Database (TCMSP) and the Traditional Chinese Medicine Information Database (TCM-ID). TCMSP is a systematic pharmacology platform designed for herbs [35]. It also serves as a systematic platform to study herbs, including the identification of compounds and compound targets. TCM-ID is an important database designed for herbal research, including the identification of herbal compounds, molecular structures, toxicity effects, etc. [36].

In addition, to identify the corresponding targets of HWD compounds, the TCMSP database and the Drugbank database were used to find potential targets. Eventually, 14 active herbal ingredients of HWD were selected (Table 1) by linking the active ingredients of HDW to the target. A total of 245 targets of HDW compounds were obtained in total.

#### 2.1.2. Pharmacokinetic Predictions

In pharmaceutical research, ADME (absorption, distribution, metabolism, and excretion) is an important pattern to identify [34]. Therefore, we employed two important ADME-related properties, namely, the oral bio-availability (OB) and drug-likeness (DL), in our study to explore the potential bio-active compounds of HWD. Ingredients with OB ≥20% and DL ≥0.1 [26,27] were selected in our study. According to the literature ursolic acid (OB 16.77% and DL 0.75) proved to be indispensable in HDW [37,38,39], therefore so we included it into our study. To be more specific, all of the HDW candidate compounds were approved by literature. The detailed information for all ingredients before screening is listed in Table S1.

#### 2.1.3. Potential Target Genes of PCa

The data for the PCa-associated target genes were obtained from two databases. The species was set to *Homo sapiens*.

(1) DisGeNET database. DisGeNET is the largest public platform which links human genes to diseases. It integrates data from scientific literature, expert curated repositories, and the genome-wide association study (GWAS) catalogues [40]. Search strategy: Download file “ALL gene-disease associations” at DisGeNET (the file contains all gene-disease associations in DisGeNET), and then set the disease name as “stage, prostate cancer”. The detailed information is listed in Table S2.

(2) Genecards. Genecards is an extensive platform which provides insight into predicted and annotated human genes. All of the gene-centric data are gathered from 150 web resources, including genetic, genomic, proteomic, transcriptomic, and functional information [41]. Search strategy: Set the keyword as “prostate cancer” and the score >30 after logging in to Genecards. The detailed information is listed in Table S3.

(3) The Online Mendelian Inheritance in Man database (OMIM). The OMIM database links and catalogues all known diseases with a genetic component and provides further references to genomic analyses of catalogued genes [42]. Search strategy: Choose gene map at the website, and then set the keyword as “prostate cancer”. The detailed information is listed in Table S4.

#### 2.1.4. Protein–Protein Interaction (PPI) Data

We obtained the PPI data from the STRING database. The STRING database defines PPI with confidence ranges for data scores (high >0.7; medium >0.4; low >0.15) [43]. In this study, we selected a confidence score of >0.7 to construct our PPI network.

### 2.2. Network Construction

Network analysis was carried out to facilitate scientific interpretation of the complicated relationships among herbs, compounds, diseases, and genes [30,33]. In the study, we generated the networks using Cytoscape (version 3.7.1) [44]. We constructed the network as follows: (1) the “HDW candidate compound target network (HDW target network)” was built by connecting the HDW compounds and the compound targets, and (2) the PPI network with complicated targets was constructed by linking targets to other human proteins interacting with it. We constructed two PPI networks, including the “HDW compound target PPI network” and the “HDW against PCa targets PPI network”. In STRING, we imported the targets by a list of names and set the organism as “homo sapiens”, set the confidence >0.7, and then exported the PPI results as a simple tabular text output (.tsv). Then, we imported the .tsv file into Cytoscape (version 3.7.1) for further analysis and applied the plugin “clustermaker” to the layout network.

### 2.3. Enrichment Analysis

#### 2.3.1. Gene Ontology (GO) Enrichment Analysis

In this study, we used the ClusterProfiler package of R3.5.0 to perform GO enrichment analysis of the targets. The higher the score, the greater the importance of the genes represented in the list [45].

#### 2.3.2. Kyoto Encyclopedia of Genes and Genomes (KEGG) Pathway Enrichment Analysis

In this study, we used the ClusterProfiler package of R3.5.0 to analyze KEGG pathway enrichment of overlapping target genes. KEGG analysis was used to explore the biological pathways and potential biological functions on the basis of the enrichment analysis of functional items [46].

## 3. Results and Discussion

In this study, we obtained a total of 14 active ingredients of HDW after ADME identification. All 14 ingredients were validated in other HDW research. Detailed information is presented in Table 1 (all Mol IDs can be tracked in the TCMSP database).

### 3.1. HDW Compound-Target Network

**Figure 1:**
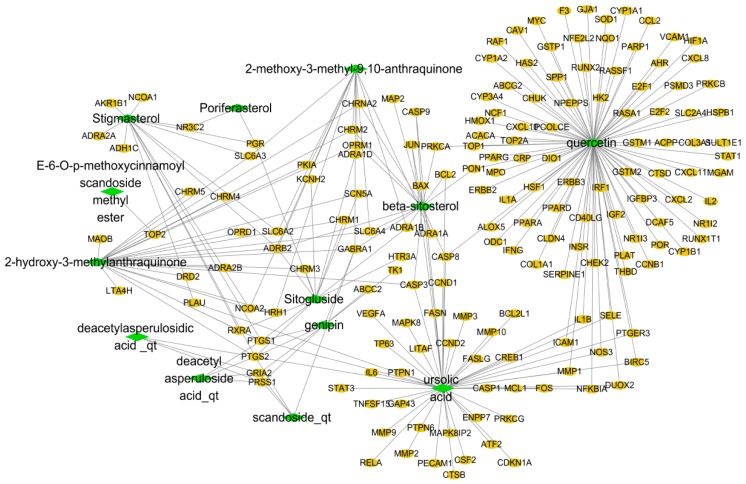
HDW compound–target network. Green represents the HDW compounds and yellow represents the targets of the HDW compounds.

The compound–target network is depicted in Figure 1, including 188 nodes and 246 edges, with a network density of 0.014 and a network diameter of 7. The detailed information of this network is shown in Table 2. The network showed that the components which connected to the most targets were quercetin (degree = 94), and ursolic acid (degree = 50), indicating that these two compounds are probably the most critical components in HDW. Quercetin is one of the most important plant flavonoids in many natural products and has been widely confirmed to have anti-cancer properties such as growth suppression, pro-apoptotic, anti-oxidant, and cell signaling effects, among others [62,63]. In addition, quercetin was confirmed to increase apoptosis and decrease colony formation via diverse effects on prostate cancer cells. A large amount of research suggests that combing quercetin with radiotherapy or chemotherapeutic agents has potential synergistic effects; moreover, quercetin can protect normal cells from side effects of radiotherapy and chemotherapy [63,64,65]. Ursolic acid is a triterpenoid found in herbs, and has been shown to inhibit prostate cancer, colon cancer, and liver cancer [66,67,68]. Studies also suggest that it can be used to prevent prostate cancer cells from uptaking glutamine when combined with resveratrol [69]. 

In addition, we found that many target genes were affected by multiple compounds. For instance, Prostaglandin-Endoperoxide Synthase 2 (PTGS2) was modulated by genipin, ursolic acid, sitogluside, etc. and Prostaglandin-Endoperoxide Synthase 1 (PTGS1) was modulated by stigmasterol, sitogluside, ursolic acid, and genipin. PTGS is well-known as the pivotal enzyme in prostaglandin biosynthesis, and it has effects both as a peroxidase and a dioxygenase [70,71]. To be more specific, inducible PTGS2 and constitutive PTGS1 are the two isozymes in PTGS, with differences concerning tissue distribution and regulation of expression [72]. PTGS1 regulates angiogenesis in endothelial cells, and the proteins it encodes have been recognized as moonlighting proteins based on their abilities to act as both cyclooxygenases and peroxidases [72,73]. In addition, PTGS2 was shown to be involved in the production of inflammatory prostaglandins by stimulatory events [72,74].

Similarly, Adrenoceptor Alpha 1B (ADRA1B), Adrenoceptor Beta 2 (ADRB2), Caspase 3 (CASP3), Caspase 8 (CASP8), Cholinergic Receptor Muscarinic 2 (CHRM2), Gamma-Aminobutyric Acid Type A Receptor Alpha1 Subunit (GABRA1), Glutamate Ionotropic Receptor AMPA Type Subunit 2 (GRIA2), Nuclear Receptor Coactivator 2 (NCOA2), Opioid Receptor Mu 1 (OPRM1), Progesterone Receptor (PGR), Serine Protease 1 (PRSS1), Retinoid X Receptor Alpha (RXRA), Solute Carrier Family 6 Member 3 (SLC6A3), and Solute Carrier Family 6 Member 4 (SLC6A4) can also be regulated by more than two ingredients. We not only obtained an approximate observation of the relationship between the bioactive compounds and the compound targets, but also discovered the potential pharmacological effects of HDW from this network. These results were consistent with other herbal research regarding network pharmacology [26,29].

### 3.2. PPI Network

The PPI data were obtained from the STRING database. STRING defines the PPI with confidence ranges for data scores (high >0.7; medium >0.4; low >0.15) [43]. Two PPI networks, including a PPI network of HDW compound targets and a PPI network of HDW compound targets against PCa, were constructed, as shown below. Due to the complexity of the original network (PPI network of HDW compound targets) obtained from the STRING database, we imported the PPI data (PPI network of HDW compounds targets) generated in STRING into Cytoscape (version 3.7.1) to reconstruct the network in order to achieve better visualization and understanding. The original STRING PPI network is presented in Figure S1. 

(1) PPI network of HDW compound targets

**Figure 2:**
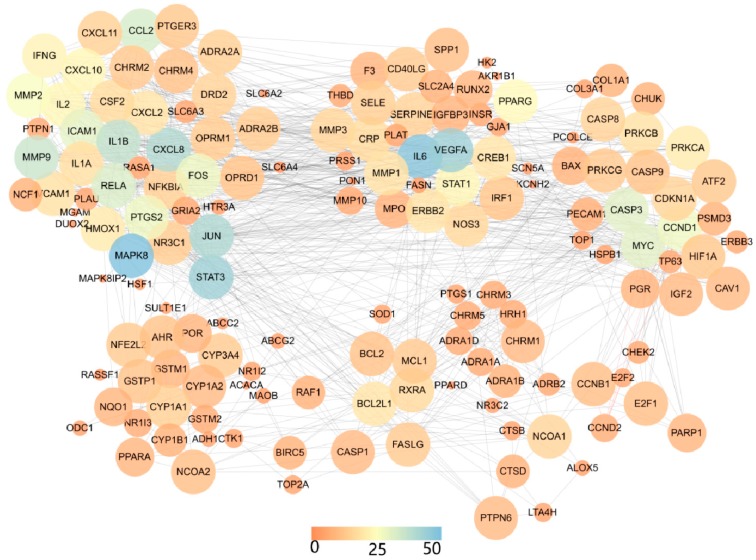
Protein–protein interaction (PPI) network of HDW compound targets. Different colors represent the degree, as the scale indicates. The size of the circle also indicates the degree.

PPI networks have been widely applied to understand many different interactions of proteins in the context of complex diseases, including breast cancer, lung cancer, bladder cancer, etc. [75]. To get the PPI network of HDW compound targets, we linked 14 active compounds to targets at the TCMSP database, and then got targets’ symbol names by uniprot. In total, 245 targets related to HDW compounds were obtained, and all of them were imported into the STRING database to generate the PPI results (settings: *Homo sapiens* and confidence >0.7). We then imported the PPI results into Cytoscape and used the plugin clustermaker to create the layout network. As Figure 2 shows, in this study, the PPI network of the HDW compound targets was built by connecting the compound targets and the interacting proteins to gain an in-depth understanding of the interaction of HDW targets at a systematic level. This PPI network included 177 nodes and 934 edges, with an average node degree of 11.81, a network diameter of 7, and an average number of 11.675 neighbors. In Figure 2, both the different colors and the size of the circles indicate the degree. In the PPI network, there was a total of 30 intersecting targets between the HDW compound targets and the PCa-related targets, namely, PTGS2, CASP3, PLAU, BCL2, BAX, CASP8, STAT3, VEGFA, CCND1, CDKN1A, MMP2, MMP9, IL6, TP63, NFKBIA, CREB1, BIRC5, RAF1, HIF1A, ERBB2, PPARG, CAV1, MYC, GSTP1, PARP1, AHR, CHEK2, RUNX2, ACPP, and IGF2. 

All 177 target degrees were calculated using this network. The 10 targets with the greatest degrees were MAPK8 (degree = 54), IL6 (degree = 48), VEGFA (degree = 44), STAT3 (degree = 44), JUN (degree = 41), CXCL8 (degree = 40), IL1B (degree = 37), MMP9 (degree = 36), CCL2 (degree = 33), and RELA (degree = 32). Further, we calculated the average “HDW compound targets and PCa-related targets” degree, with the value resulting in 18.86, which was 7.05 more than the overall average node degree. In addition, the IL6 (degree = 48), VEGFA (degree = 44), and STAT3 (degree = 44) of the “HDW compound targets and PCa-related targets” all appeared in the top 10 degrees in the overall PPI network. 

IL6, a glycogen composed of 184 amino acids, is a multi-functional cell cytokine that affects cancer cell activity [76]. It has been widely shown to influence tumor growth, micro environment immunomodulation, and malignant differentiation of cancer cells [77]. These effects come about via several pathways, of which the signal transducer and the transcription activator play the most important role. Moreover, several studies found that IL6 was overexpressed in diverse cancer cells and IL6 levels were elevated in late-stage cancer [78,79]. VEGFA, also known as VEGF-A, is a homodimeric glycoprotein that stimulates vascular endothelial cell proliferation, growth, and survival [22]. VEGFA is a key mediator of tumor angiogenesis, and its expression is regulated by oncogenes, various growth factors, and hypoxia [80]. It is known that angiogenesis is essential for the growth and development of cancer, and it plays an important role in the metastasis, occurrence, and proliferation of prostate cancer as well [81]. Tumor-derived VEGF can cause an "angiogenic switch", which creates a new vascular system inside and around the tumor, thereby allowing tumor cells to proliferate. Therefore, the effect of VEGF in the production of the tumor vascular system makes it an important target in anti-cancer therapies [81]. In addition to the two important HDW compound targets, other targets, such as MAPK8, STAT3 , JUN, CXCL8, IL1B, MMP9, CCL2, RELA, PTGS2, CASP3, PLAU, BCL2, BAX, CASP8,CCND1, CDKN1A, TP63, NFKBIA, CREB1, BIRC5, RAF1, HIF1A, ERBB2, PPARG, CAV1, MYC, GSTP1, PARP1, AHR, CHEK2, RUNX2, and ACPP, can influence cancer cell activity. These findings showed that HDW had a significant effect on PCa by influencing the entire biological network, which consisted of 30 common targets. 

(2) PPI network of HWD compound targets against PCa

**Figure 3:**
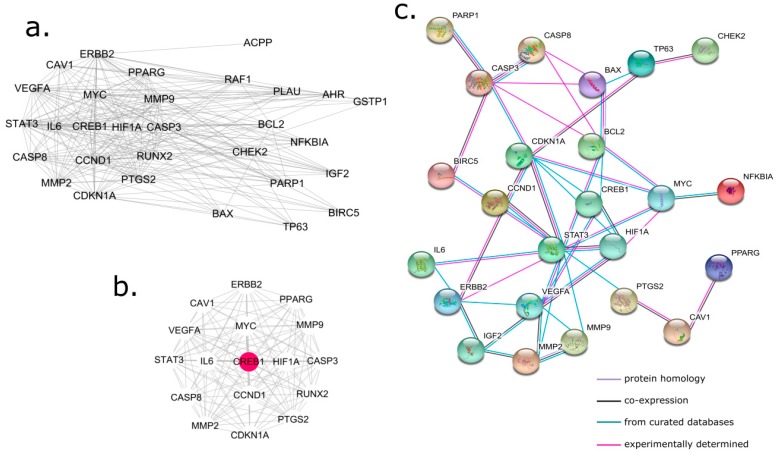
PPI network of HWD compound targets against prostate cancer (PCa). (**a**) The PPI network constructed using Cytoscape (version 3.7.1); (**b**) the cluster generated from (a), where the red target represents CREB1, the seed in this cluster; (**c**) the original PPI data generated from the STRING database showing the detailed interactions of the targets.

To explore the potential mechanisms of HDW as a therapy against PCa, a PPI network of the HWD compound targets against PCa was constructed by connecting the HDW compound targets and the PCa targets. Further cluster analysis was done by using MCODE [82], a tool in Cytoscape (version 3.7.1) to generate clusters in the network (Figure 3b). As shown in Figure 3a, this PPI network consisted of 30 nodes and 227 edges. The clustering coefficient was 0.766 and the network diameter was 3. The average degree in Figure 3a was 15.13 and there were 15 target degrees greater than the average. These targets were MYC, CCND1, STAT3, CASP3, ERBB2, VEGFA, IL6, CDKN1A, MMP9, PTGS2, HIF1A, MMP2, CASP8, PPARG, and CREB1. As shown in Figure 3b, the cluster consisted of 17 nodes and 125 edges. The clustering coefficient was 0.938 and the network density was 0.919. The red circle in Figure 3b, CREB1, was the seed in this cluster, indicating that CREB1 played an important role in connecting other nodes in this PPI network. It is well-known that CREB1, a member of the leucine zipper family of DNA binding proteins, is a cancer-related gene [83]. Recent studies showed that CREB1 overexpression occurred in prostate cancer tissues [84], acute leukemia [85], and non-small-cell lung cancer [86]. In addition, several pieces of research suggested that CREB1 may be a promising target for tumor therapy, since the downregulation of CREB1 results in the inhibition of proliferation and induction in several different cancer cell lines [87,88].

The cluster (Figure 3b) shows that CREB1, as the seed in the cluster, interacted with other HDW targets. Figure 3c was automatically generated from the STRING database, which intuitively demonstrates protein homology and co-expression information and provides information regarding the source of the interactions. 

### 3.3. GO Enrichment

**Figure 4:**
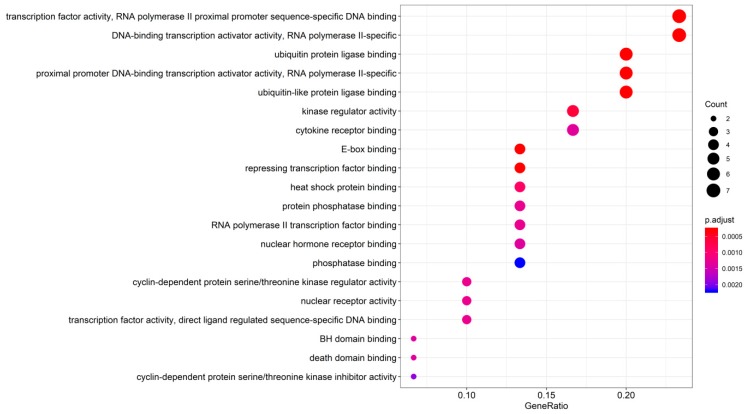
Gene enrichment (GO) analysis for the 30 shared HDW compound targets/PCa-related targets. The color represents the different adjusted *p*-values (<0.05), while the size of the circle represents the count.

**Figure 5:**
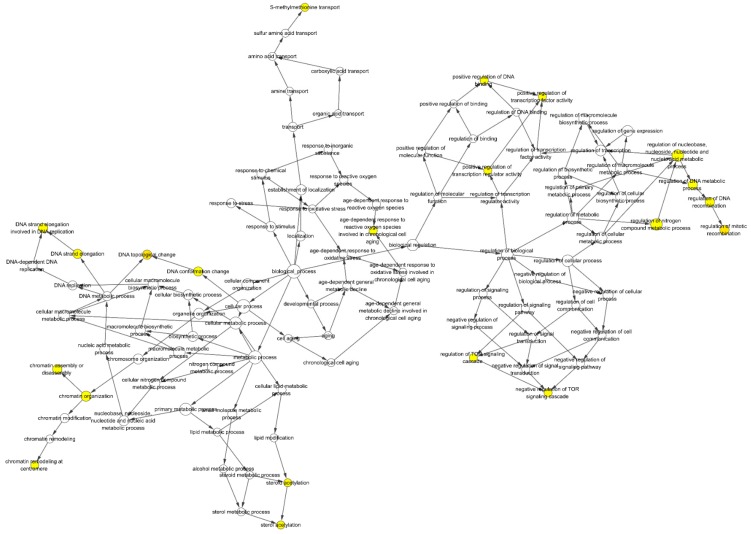
The biological process of HDW. Yellow circles represent the biological processes with adjusted *p*-values <0.05.

To further explore the multiple mechanisms of HDW as a therapy against PCa, we performed GO enrichment analysis (molecular function in Figure 4 and biological processes in Figure 5) [45] on the 30 common targets shared by the HDW compound targets and the PCa-related targets. To be more specific, the 30 common targets are as follows: PTGS2, CASP3, FF0C;BCL2, BAX, CASP8, STAT3, VEGFA, CCND1, CDKN1A, MMP2, MMP9, IL6, TP63, NFKBIA, CREB1, BIRC5, RAF1, HIF1A, ERBB2, PPARG, CAV1, MYC, GSTP1, PARP1, AHR, CHEK2, RUNX2, ACPP, and IGF2. The top 20 significantly enriched GO targets are presented (adjusted *p*-value < 0.05) in Figure 4. The top five GO enrichment targets included (1) transcription factor activity, RNA polymerase II proximal promoter sequence-specific DNA binding (GO:0000982); (2) DNA-binding transcription activator activity, RNA polymerase II-specific (GO:0001228); (3) ubiquitin protein ligase binding (GO:0031625); (4) proximal promoter DNA-binding transcription activator activity, RNA polymerase II-specific (GO:0001077); and (5) ubiquitin-like protein ligase binding (GO:0044389). Detailed GO enrichment information is shown in Table 3. To better understand the biological processes [89] of the HDW compound targets, we performed and visualized analyses of the biological processes of the HDW compound targets. As Figure 5 shows, yellow circles represent the biological processes with adjusted *p*-values of <0.05. After corrections, 21 statistically significant biological processes were obtained. According to their node size, the five most obvious biological processes were nucleobase, nucleoside, nucleotide, and nucleic acid metabolic process regulation, nitrogen compound metabolic process regulation, chromatin organization, DNA topological change, mitotic recombination regulation, and DNA recombination regulation. Detailed descriptions for these biological processes are presented in Table S1. 

By analyzing the molecular functions and biological processes, we suggest that HDW could have pharmacological effects on PCa [21]. Our GO enrichment showed that HDW was strongly related to translesion synthesis, unfolded protein binding, regulation of mitotic recombination, phosphatidylinositol and its kinase-mediated signaling, nucleotide excision repair, regulation of DNA recombination, and DNA topological change [26]. 

### 3.4. KEGG Enrichment

**Figure 6:**
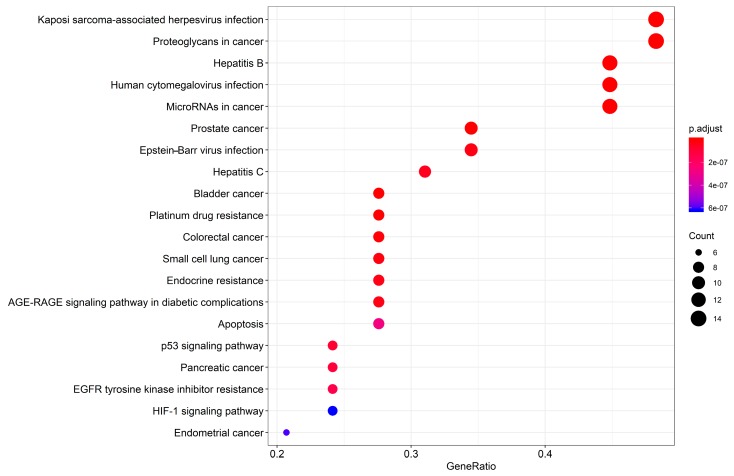
KEGG enrichment analysis for the 30 shared HDW compound targets/PCa-related targets. The color represents the different adjusted *p*-value < 0.05, while the size of circle represents the count. Abbreviations: EGFR, epidermal growth factor receptor; HIF, hypoxia-inducible factor.

As is shown in Figure 6, we further performed KEGG [46] enrichment analysis on the 30 common targets shared by the HDW compound targets and the PCa-related targets. We obtained 87 pathways in total which belonged to several categories, including human diseases, environmental information, organismal systems, and cellular processes, among others, of which the top 20 significantly enriched KEGG targets are presented (adjusted *p*-value < 0.05) in Figure 6. In the cancer-related disease, prostate cancer (hsa05215), bladder cancer (hsa05219), colorectal cancer (hsa05210), and small cell lung cancer (hsa05222) data were processed using KEGG enrichment analysis. Detailed KEGG information is shown in Table 4. This result indicated that HDW had the potential to affect a diverse range of cancers, such as prostate cancer, bladder cancer, colorectal cancer, and small cell lung cancer, which was confirmed by other researchers [18,26]. This KEGG enrichment result showed that HDW was highly involved in the regulation of angiogenesis, cell differentiation, migration, apoptosis, invasion, and proliferation [20,23]. Therefore, we speculate that the underlying mechanism of HDW against PCa may be due to its coordinated regulation of several cancer-related pathways [26].

## 4. Conclusions

Up to now, although many cancer studies confirmed that HDW exhibited noticeable anti-tumor effects, the potential mechanisms of its anti-tumor effects have not yet been fully explored. Network pharmacology emphasizes the integration of bioinformatics, systems biology, and pharmacology, which not only interprets the complicated interactions between herbs and diseases at a systematic level, but also conforms to the systematic and holistic perspective of the TCM theory. To better understand the pharmacological mechanisms of HDW as a therapy for PCa, in this study, we applied the network pharmacology approach to explore the potential mechanisms of HDW as a therapy against PCa by compound–target network construction, PPI network analysis, GO enrichment analysis, and KEGG enrichment analysis. We employed two OB and DL to explore the potential bio-active compounds of HWD. Up to now, the researches on the pharmacokinetics of HDW are scare [90,91]. Ganbold et al. [92] found that HDW has good permeability in vitro with no cytotoxic effect by investigating the OB of HDW by production of post-absorption samples using the Caco-2 cell model. In our study, we obtained 14 active compounds from HDW and 295 potential targets in total, and demonstrated a synergistic herb strategy featuring multi-component, multi-target, and multi-pathway characteristics. The compound–target network indicated that quercetin and ursolic acid served as the main ingredients in HDW. Furthermore, the PPI network demonstrated information regarding protein homology and co-expression, and also provided information concerning the source of the interactions. Our PPI analysis indicated that HDW had a significant effect on PCa by influencing the entire biological network, including targets such as MAPK8, IL6, VEGFA, STAT3, JUN, CXCL8, IL1B, MMP9, CCL2, RELA, and CREB1. The PPI cluster demonstrated that CREB1 was the seed, indicating that CREB1 played an important role in connecting other nodes in this PPI network. Thirdly, the enrichment showed that HDW was strongly related to translesion synthesis, unfolded protein binding, regulation of mitotic recombination, phosphatidylinositol and its kinase-mediated signaling, nucleotide excision repair, regulation of DNA recombination, and DNA topological change. The enrichment results also presented that the underlying mechanism of HDW against PCa may be due to its coordinated regulation of several cancer-related pathways, such as angiogenesis, cell differentiation, migration, apoptosis, invasion, and proliferation, among others. 

In summary, this study applied a network approach documenting how HDW compounds alter different pathways against PCa, which is supplementary to other studies on drugs against PCa. Moreover, we demonstrated that HDW substantially influenced a number of PCa-related targets, a finding which was consistent with present cancer research trends showing that PCa can be attributed to the gradual accumulation of distinct genome modifications in tumor cells. We fully expect that our research can help to promote the employment of network pharmacology in uncovering the potential mechanisms of anti-cancer herbs, and provide clues to assess the synergy of herbs in the treatment of other complex diseases. However, from a critical point of view, there are limitations in this study. Since this study was based on data analysis, further experiments (Western blot or real-time PCR analysis) are needed to validate our findings. In addition, although rare literature reported the cytotoxicity of HDW, further experiments on normal epithelial cells are needed to validate the potential cytotoxicity. Meanwhile, pharmacokinetics researches are needed to illustrate the characteristics of HDW against PCa.

## Figures and Tables

**Table 1:** Active ingredients of *Hedyotis diffusa* Willd (HDW).

Mol ID	Mol Name	2D Structure	OB (%)	DL	Reference
MOL001649	2-hydroxy-3-methylanthraquinone	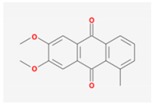	26.09	0.18	[47,48]
MOL001650	*E*-6-*O*-*p*-methoxycinnamoyl scandoside methyl ester	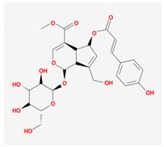	27.12	0.81	[49,50,51]
MOL001657	scandoside_qt	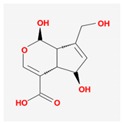	30.02	0.1	[52]
MOL001659	Poriferasterol	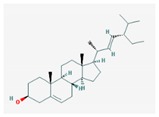	43.83	0.76	[19]
MOL001667	deacetyl asperuloside acid_qt	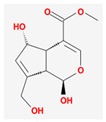	62.46	0.11	[53,54]
MOL001670	2-methoxy-3-methyl-9,10-anthraquinone	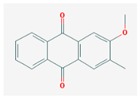	37.83	0.21	[19]
MOL000449	Stigmasterol	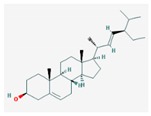	43.83	0.76	[19,55,56]
MOL000357	Sitogluside	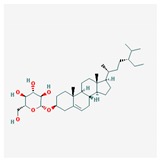	20.63	0.62	[57]
MOL000358	beta-sitosterol	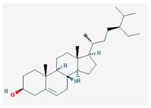	36.91	0.75	[58]
MOL001646	2,3-dimethoxy-6-methyanthraquinone	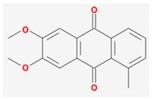	34.86	0.26	[59]
MOL000511	ursolic acid	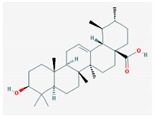	16.77	0.75	[37,38,39]
MOL000665	deacetylasperulosidic acid _qt	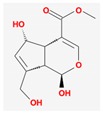	30.29	0.10	[52]
MOL000098	quercetin	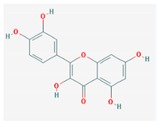	46.43	0.28	[60]
MOL001648	genipin	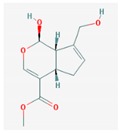	26.06	0.1	[61]

Abbreviations: OB, oral bio-availability; DL: drug-likeness; Mol: molecular.

**Table 2:** HDW compound–candidate target network parameters.

Network Parameters	Values
Number of nodes	188
Network density	0.014
Network diameter	7
Network heterogeneity	3.056
Average number of neighbors	2.317
Characteristic path length	3.518
Shortest paths	33,676 (95%)
Network centralization	0.494

**Table 3:** Enrichment results.

ID	Description	Count	Adjust *p*-Value
GO:0000982	Transcription factor activity, RNA polymerase II proximal promoter sequence-specific DNA binding	7	0.0003
GO:0001228	DNA-binding transcription activator activity, RNA polymerase II-specific	7	0.0003
GO:0031625	Ubiquitin protein ligase binding	6	0.0003
GO:0001077	Proximal promoter DNA-binding transcription activator activity, RNA polymerase II-specific	6	0.0003
GO:0044389	Ubiquitin-like protein ligase binding	6	0.0003
GO:0019207	Kinase regulator activity	5	0.0006
GO:0005126	Cytokine receptor binding	5	0.0013
GO:0070888	E-box binding	4	0.0003
GO:0070491	Repressing transcription factor binding	4	0.0003
GO:0031072	Heat shock protein binding	4	0.0009
GO:0019903	Protein phosphatase binding	4	0.0013
GO:0001085	RNA polymerase II transcription factor binding	4	0.0013
GO:0035257	Nuclear hormone receptor binding	4	0.0014
GO:0019902	Phosphatase binding	4	0.0022
GO:0016538	Cyclin-dependent protein serine/threonine kinase regulator activity	3	0.0013
GO:0004879	Nuclear receptor activity	3	0.0013
GO:0098531	Transcription factor activity, direct ligand regulated sequence-specific DNA binding	3	0.0013
GO:0051400	Bcl-2 Homology (BH) domain binding	2	0.0014
GO:0070513	Death domain binding	2	0.0014
GO:0004861	Cyclin-dependent protein serine/threonine kinase inhibitor activity	2	0.0019

**Table 4:** Kyoto Encyclopedia of Genes and Genomes (KEGG) enrichment results.

ID	Description	Count	Adjusted *p*-Value
hsa05167	Kaposi sarcoma-associated herpesvirus infection	14	0.0000
hsa05205	Proteoglycans in cancer	14	0.0000
hsa05161	Hepatitis B	13	0.0000
hsa05163	Human cytomegalovirus infection	13	0.0000
hsa05206	MicroRNAs in cancer	13	0.0000
hsa05215	Prostate cancer	10	0.0000
hsa05169	Epstein–Barr virus infection	10	0.0000
hsa04151	PI3K-Akt signaling pathway	10	0.0000
hsa05160	Hepatitis C	9	0.0000
hsa05165	Human papillomavirus infection	9	0.0000
hsa05219	Bladder cancer	8	0.0000
hsa01524	Platinum drug resistance	8	0.0000
hsa05210	Colorectal cancer	8	0.0000
hsa05222	Small cell lung cancer	8	0.0000
hsa01522	Endocrine resistance	8	0.0000
hsa04933	AGE-RAGE signaling pathway in diabetic complications	8	0.0000
hsa04210	Apoptosis	8	0.0000
hsa05202	Transcriptional misregulation in cancer	8	0.0000
hsa05203	Viral carcinogenesis	8	0.0000
hsa05166	Human T-cell leukemia virus 1 infection	8	0.0000

## Supplementary Materials

The following are available online at https://www.mdpi.com/2218-273X/9/10/591/s1, Figure S1: PPI network of HDW compounds targets from the STRING database, Table S1: All the HDW compounds before screening, Table S2: PCa related gene results in DisGeNet, Table S3: PCa related gene results in Genecards, Table S4: PCa related gene results in OMIM, Table S5: The data used in PPI network of HDW compound targets, Table S6: The data used in the PPI network of HWD compound targets against PCa, Table S7: Detailed biological processes of HDW.

## Abbreviations

HDW	*Hedyotis diffusa* Willd
PCa	Prostate cancer
PPI	Protein–protein interaction
RP	Radical prostatectomy
TCM	Traditional Chinese Medicine
OB	Oral bio-availability
DL	Drug-likeness
TCMSP	Traditional Chinese Medicine Systems Pharmacology Database
TCM-ID	Traditional Chinese Medicine Information Database
ADME	Absorption, distribution, metabolism, and excretion
GWAS	Genome-wide association study
OMIM	Online Mendelian Inheritance in Man
GO	Gene Ontology
KEGG	Kyoto Encyclopedia of Genes and Genomes
