# Supplementary material for: Investigating the Multi-Target Pharmacological Mechanism of Hedyotis diffusa Willd Acting on Prostate Cancer: A Network Pharmacology Approach
Source: Biomolecules. 2019 Oct 9;9(10):591. doi: 10.3390/biom9100591 (PMC6843553; doi:10.3390/biom9100591)
Supplement: Supplementary file 1 [file biomolecules-09-00591-s001.pdf]

Supplementary material:

Supplementary Figure1	PPI network of HDW compounds targets from the STRING database
Supplementary Table 1	All the HDW compounds before screening
Supplementary Table 2	PCa related gene results in DisGeNet
Supplementary Table 3	PCa related gene results in Genecards
Supplementary Table 4	PCa related gene results in OMIM
Supplementary Table 5	The data used in PPI network of HDW compound targets
Supplementary Table 6	The data used in the PPI network of HWD compound targets against PCa
Supplementary Table 7	Detailed biological processes of HDW

Supplementary Figure1:PPI network of HDW compounds targets from the STRING database.

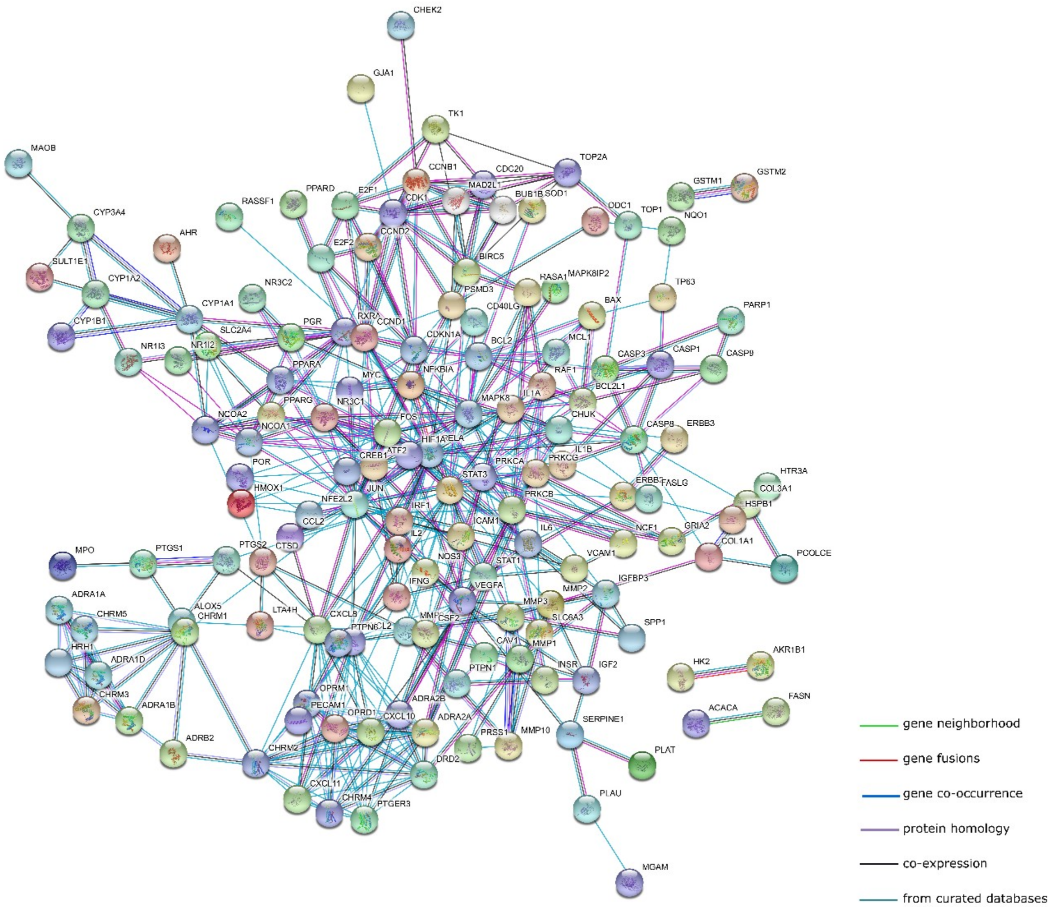

Supplementary Table 1. All the HDW compounds before screening

Mol ID	MolName	OB (%)	DL
MOL001646	2,3-dimethoxy-6-methyanthraquinone	34.86	0.26
MOL004557	geniposide	14.64	0.44
MOL001648	genipin	26.06	0.1
MOL001649	2-hydroxy-3-methylanthraquinone	26.09	0.18
MOL001650	E-6-O-p-methoxycinnamoyl scandoside methyl ester	27.12	0.81
MOL001651	E-6-O-p-methoxycinnamoyl scandoside methyl ester_qt	14.44	0.51
MOL001652	1H-2,6-dioxacyclopent(cd)inden-1-one, 4-((acetyloxy)methyl)-5-(beta-D-glucopyranosyloxy)-2a,4a,5,7b-tetrahydro-, (2aS-(2aalpha,5alpha,7balpha))-	26.43	0.71
MOL001653	asperuloside_qt	6.86	0.17
MOL001654	Oleanolic acid-28-O-beta-D-glucopyranoside	11.48	0.41
MOL001655	oleanolic acid-3-O-β-D-glucuronopyranoside_qt	17.93	0.76
MOL001656	(1S,4aS,5R,7aS)-5-hydroxy-7-methylol-1-[(2S,3R,4S,5S,6R)-3,4,5-trihydroxy-6-methylol-tetrahydropyran-2-yl]oxy-1,4a,5,7a-tetrahydrocyclopenta[d]pyran-4-carboxylic acid	3.55	0.45

MOL001657	scandoside_qt	30.02	0.1
MOL001658	3'-Hydroxyanethole	30.01	0.04
MOL001659	Poriferasterol	43.83	0.76
MOL001660	p-MCA	31	0.05
MOL001661	Scandoside methyl ester	11.24	0.48
MOL001662	scandoside_qt	14.41	0.11
MOL001663	(4aS,6aR,6aS,6bR,8aR,10R,12aR,14bS)-10-hydroxy-2,2,6a,6b,9,9,12a-heptamethyl-1,3,4,5,6,6a,7,8,8a,10,11,12,13,14b-tetradecahydropicene-4a-carboxylic acid	32.03	0.76
MOL001664	deacetylasperulosidic acid	3.42	0.45
MOL001665	deacetylasperulosidic acid _qt	30.29	0.1
MOL001666	Deacetyl asperulosidic acid methyl ester	4.29	0.48
MOL001667	deacetyl asperuloside acid_qt	62.46	0.11
MOL001668	Geniposidic acid	19.59	0.41
MOL001669	geniposidie acid_qt	30.96	0.09
MOL001670	2-methoxy-3-methyl-9,10-anthraquinone	37.83	0.21
MOL001671	Digitolutein	19.36	0.23
MOL000449	Stigmasterol	43.83	0.76
MOL000346	succinic acid	29.62	0.01
MOL000357	Sitogluside	20.63	0.62
MOL000358	beta-sitosterol	36.91	0.75
MOL000360	FER	39.56	0.06
MOL000040	Scopoletol	27.77	0.08
MOL000415	rutin	3.2	0.68
MOL000511	ursolic acid	16.77	0.75
MOL000628	darutoside	21.32	0.63
MOL000771	p-coumaric acid	43.29	0.04
MOL000098	quercetin	46.43	0.28

Supplementary Table 2. PCa related gene results in DisGeNet

Disease name	GeneID	Symbol	Gene Name
stage, prostate cancer	367	AR	androgen receptor
stage, prostate cancer	354	KLK3	kallikrein related peptidase 3
stage, prostate cancer	2078	ERG	ERG, ETS transcription factor
stage, prostate cancer	7157	TP53	tumor protein p53
stage, prostate cancer	3569	IL6	interleukin 6
stage, prostate cancer	7040	TGFB1	transforming growth factor beta 1
stage, prostate cancer	145957	NRG4	neuregulin 4
stage, prostate cancer	7098	TLR3	toll like receptor 3
stage, prostate cancer	6755	SSTR5	somatostatin receptor 5
stage, prostate cancer	5743	PTGS2	prostaglandin-endoperoxide synthase 2
stage, prostate cancer	5627	PROS1	protein S
stage, prostate cancer	5324	PLAG1	PLAG1 zinc finger

stage, prostate cancer	5058	PAK1	p21 (RAC1) activated kinase 1
stage, prostate cancer	7227	TRPS1	transcriptional repressor GATA binding 1
stage, prostate cancer	7422	VEGFA	vascular endothelial growth factor A
stage, prostate cancer	7432	VIP	vasoactive intestinal peptide
stage, prostate cancer	116984	ARAP2	ArfGAP with RhoGAP domain, ankyrin repeat and PH domain 2
stage, prostate cancer	58515	SELENOK	selenoprotein K
stage, prostate cancer	51203	NUSAP1	nucleolar and spindle associated protein 1
stage, prostate cancer	51181	DCXR	dicarbonyl and L-xylulose reductase
stage, prostate cancer	29968	PSAT1	phosphoserine aminotransferase 1
stage, prostate cancer	9622	KLK4	kallikrein related peptidase 4
stage, prostate cancer	9566	HPCX	hereditary prostate cancer, X-linked
stage, prostate cancer	9520	NPEPPS	aminopeptidase puromycin sensitive
stage, prostate cancer	8667	EIF3H	eukaryotic translation initiation factor 3 subunit H
stage, prostate cancer	4780	NFE2L2	nuclear factor, erythroid 2 like 2
stage, prostate cancer	4609	MYC	MYC proto-oncogene, bHLH transcription factor
stage, prostate cancer	4481	MSR1	macrophage scavenger receptor 1
stage, prostate cancer	2152	F3	coagulation factor III, tissue factor
stage, prostate cancer	2146	EZH2	enhancer of zeste 2 polycomb repressive complex 2 subunit
stage, prostate cancer	1958	EGR1	early growth response 1
stage, prostate cancer	1950	EGF	epidermal growth factor
stage, prostate cancer	1586	CYP17A1	cytochrome P450 family 17 subfamily A member 1
stage, prostate cancer	1577	CYP3A5	cytochrome P450 family 3 subfamily A member 5
stage, prostate cancer	820	CAMP	cathelicidin antimicrobial peptide
stage, prostate cancer	472	ATM	ATM serine/threonine kinase
stage, prostate cancer	347	APOD	apolipoprotein D
stage, prostate cancer	2324	FLT4	fms related tyrosine kinase 4
stage, prostate cancer	2551	GABPA	GA binding protein transcription factor subunit alpha
stage, prostate cancer	4316	MMP7	matrix metallopeptidase 7
stage, prostate cancer	4233	MET	MET proto-oncogene, receptor tyrosine kinase
stage, prostate cancer	4072	EPCAM	epithelial cell adhesion molecule
stage, prostate cancer	3732	CD82	CD82 molecule
stage, prostate cancer	3600	IL15	interleukin 15
stage, prostate cancer	3574	IL7	interleukin 7
stage, prostate cancer	3479	IGF1	insulin like growth factor 1
stage, prostate cancer	2950	GSTP1	glutathione S-transferase pi 1
stage, prostate cancer	2852	GPER1	G protein-coupled estrogen receptor 1
stage, prostate cancer	216	ALDH1A1	aldehyde dehydrogenase 1 family member A1

Supplementary Table 3.PCa related gene results in Genecards

Symbol	GeneCards Link
BRCA2	https://www.genecards.org/cgi-bin/carddisp.pl?gene=BRCA2
BRCA1	https://www.genecards.org/cgi-bin/carddisp.pl?gene=BRCA1
TP53	https://www.genecards.org/cgi-bin/carddisp.pl?gene=TP53

PTEN	https://www.genecards.org/cgi-bin/carddisp.pl?gene=PTEN
CDH1	https://www.genecards.org/cgi-bin/carddisp.pl?gene=CDH1
CHEK2	https://www.genecards.org/cgi-bin/carddisp.pl?gene=CHEK2
AR	https://www.genecards.org/cgi-bin/carddisp.pl?gene=AR
EGFR	https://www.genecards.org/cgi-bin/carddisp.pl?gene=EGFR
ATM	https://www.genecards.org/cgi-bin/carddisp.pl?gene=ATM
ERBB2	https://www.genecards.org/cgi-bin/carddisp.pl?gene=ERBB2
CTNNB1	https://www.genecards.org/cgi-bin/carddisp.pl?gene=CTNNB1
PIK3CA	https://www.genecards.org/cgi-bin/carddisp.pl?gene=PIK3CA
KLK3	https://www.genecards.org/cgi-bin/carddisp.pl?gene=KLK3
AKT1	https://www.genecards.org/cgi-bin/carddisp.pl?gene=AKT1
NBN	https://www.genecards.org/cgi-bin/carddisp.pl?gene=NBN
KRAS	https://www.genecards.org/cgi-bin/carddisp.pl?gene=KRAS
MLH1	https://www.genecards.org/cgi-bin/carddisp.pl?gene=MLH1
CDKN2A	https://www.genecards.org/cgi-bin/carddisp.pl?gene=CDKN2A
CCND1	https://www.genecards.org/cgi-bin/carddisp.pl?gene=CCND1
APC	https://www.genecards.org/cgi-bin/carddisp.pl?gene=APC
VEGFA	https://www.genecards.org/cgi-bin/carddisp.pl?gene=VEGFA
MYC	https://www.genecards.org/cgi-bin/carddisp.pl?gene=MYC
ESR1	https://www.genecards.org/cgi-bin/carddisp.pl?gene=ESR1
MSH2	https://www.genecards.org/cgi-bin/carddisp.pl?gene=MSH2
MSH6	https://www.genecards.org/cgi-bin/carddisp.pl?gene=MSH6
CDKN1B	https://www.genecards.org/cgi-bin/carddisp.pl?gene=CDKN1B
HRAS	https://www.genecards.org/cgi-bin/carddisp.pl?gene=HRAS
CDKN1A	https://www.genecards.org/cgi-bin/carddisp.pl?gene=CDKN1A
RB1	https://www.genecards.org/cgi-bin/carddisp.pl?gene=RB1
TERT	https://www.genecards.org/cgi-bin/carddisp.pl?gene=TERT
MET	https://www.genecards.org/cgi-bin/carddisp.pl?gene=MET
STAT3	https://www.genecards.org/cgi-bin/carddisp.pl?gene=STAT3
MDM2	https://www.genecards.org/cgi-bin/carddisp.pl?gene=MDM2
TGFB1	https://www.genecards.org/cgi-bin/carddisp.pl?gene=TGFB1
SMAD4	https://www.genecards.org/cgi-bin/carddisp.pl?gene=SMAD4
BRAF	https://www.genecards.org/cgi-bin/carddisp.pl?gene=BRAF
CDK4	https://www.genecards.org/cgi-bin/carddisp.pl?gene=CDK4
BAX	https://www.genecards.org/cgi-bin/carddisp.pl?gene=BAX
PALB2	https://www.genecards.org/cgi-bin/carddisp.pl?gene=PALB2
RNASEL	https://www.genecards.org/cgi-bin/carddisp.pl?gene=RNASEL
FGFR2	https://www.genecards.org/cgi-bin/carddisp.pl?gene=FGFR2
MTOR	https://www.genecards.org/cgi-bin/carddisp.pl?gene=MTOR
MSMB	https://www.genecards.org/cgi-bin/carddisp.pl?gene=MSMB
PLAU	https://www.genecards.org/cgi-bin/carddisp.pl?gene=PLAU
ESR2	https://www.genecards.org/cgi-bin/carddisp.pl?gene=ESR2
RAD50	https://www.genecards.org/cgi-bin/carddisp.pl?gene=RAD50
CASP8	https://www.genecards.org/cgi-bin/carddisp.pl?gene=CASP8

KLF6	https://www.genecards.org/cgi-bin/carddisp.pl?gene=KLF6
SRC	https://www.genecards.org/cgi-bin/carddisp.pl?gene=SRC
ELAC2	https://www.genecards.org/cgi-bin/carddisp.pl?gene=ELAC2
HOXB13	https://www.genecards.org/cgi-bin/carddisp.pl?gene=HOXB13
PMS2	https://www.genecards.org/cgi-bin/carddisp.pl?gene=PMS2
MIR21	https://www.genecards.org/cgi-bin/carddisp.pl?gene=MIR21
H19	https://www.genecards.org/cgi-bin/carddisp.pl?gene=H19
SRD5A2	https://www.genecards.org/cgi-bin/carddisp.pl?gene=SRD5A2
BRIP1	https://www.genecards.org/cgi-bin/carddisp.pl?gene=BRIP1
IGF2	https://www.genecards.org/cgi-bin/carddisp.pl?gene=IGF2
CYP17A1	https://www.genecards.org/cgi-bin/carddisp.pl?gene=CYP17A1
MAP2K1	https://www.genecards.org/cgi-bin/carddisp.pl?gene=MAP2K1
IL6	https://www.genecards.org/cgi-bin/carddisp.pl?gene=IL6
ACPP	https://www.genecards.org/cgi-bin/carddisp.pl?gene=ACPP
FOLH1	https://www.genecards.org/cgi-bin/carddisp.pl?gene=FOLH1
BCL2	https://www.genecards.org/cgi-bin/carddisp.pl?gene=BCL2
EPHB2	https://www.genecards.org/cgi-bin/carddisp.pl?gene=EPHB2
FGFR1	https://www.genecards.org/cgi-bin/carddisp.pl?gene=FGFR1
ERG	https://www.genecards.org/cgi-bin/carddisp.pl?gene=ERG
MSR1	https://www.genecards.org/cgi-bin/carddisp.pl?gene=MSR1
TNF	https://www.genecards.org/cgi-bin/carddisp.pl?gene=TNF
FAS	https://www.genecards.org/cgi-bin/carddisp.pl?gene=FAS
EP300	https://www.genecards.org/cgi-bin/carddisp.pl?gene=EP300
MXI1	https://www.genecards.org/cgi-bin/carddisp.pl?gene=MXI1
RAF1	https://www.genecards.org/cgi-bin/carddisp.pl?gene=RAF1
HNF1B	https://www.genecards.org/cgi-bin/carddisp.pl?gene=HNF1B
VDR	https://www.genecards.org/cgi-bin/carddisp.pl?gene=VDR
PDGFRB	https://www.genecards.org/cgi-bin/carddisp.pl?gene=PDGFRB
FGFR4	https://www.genecards.org/cgi-bin/carddisp.pl?gene=FGFR4
MIR221	https://www.genecards.org/cgi-bin/carddisp.pl?gene=MIR221
AKT2	https://www.genecards.org/cgi-bin/carddisp.pl?gene=AKT2
GSTP1	https://www.genecards.org/cgi-bin/carddisp.pl?gene=GSTP1
BARD1	https://www.genecards.org/cgi-bin/carddisp.pl?gene=BARD1
RAD51C	https://www.genecards.org/cgi-bin/carddisp.pl?gene=RAD51C
EGF	https://www.genecards.org/cgi-bin/carddisp.pl?gene=EGF
MIR34A	https://www.genecards.org/cgi-bin/carddisp.pl?gene=MIR34A
MIR145	https://www.genecards.org/cgi-bin/carddisp.pl?gene=MIR145
NF1	https://www.genecards.org/cgi-bin/carddisp.pl?gene=NF1
PTGS2	https://www.genecards.org/cgi-bin/carddisp.pl?gene=PTGS2
KIT	https://www.genecards.org/cgi-bin/carddisp.pl?gene=KIT
TNFRSF10B	https://www.genecards.org/cgi-bin/carddisp.pl?gene=TNFRSF10B
MMP9	https://www.genecards.org/cgi-bin/carddisp.pl?gene=MMP9
PSCA	https://www.genecards.org/cgi-bin/carddisp.pl?gene=PSCA
TGFBR2	https://www.genecards.org/cgi-bin/carddisp.pl?gene=TGFBR2

MIR143	https://www.genecards.org/cgi-bin/carddisp.pl?gene=MIR143
RAD51D	https://www.genecards.org/cgi-bin/carddisp.pl?gene=RAD51D
CDKN3	https://www.genecards.org/cgi-bin/carddisp.pl?gene=CDKN3
MIR222	https://www.genecards.org/cgi-bin/carddisp.pl?gene=MIR222
MIR205	https://www.genecards.org/cgi-bin/carddisp.pl?gene=MIR205
MIR141	https://www.genecards.org/cgi-bin/carddisp.pl?gene=MIR141
CASP3	https://www.genecards.org/cgi-bin/carddisp.pl?gene=CASP3
MIR146A	https://www.genecards.org/cgi-bin/carddisp.pl?gene=MIR146A
MIR203A	https://www.genecards.org/cgi-bin/carddisp.pl?gene=MIR203A
EHBP1	https://www.genecards.org/cgi-bin/carddisp.pl?gene=EHBP1
KRT8	https://www.genecards.org/cgi-bin/carddisp.pl?gene=KRT8
MAD1L1	https://www.genecards.org/cgi-bin/carddisp.pl?gene=MAD1L1
FOXO1	https://www.genecards.org/cgi-bin/carddisp.pl?gene=FOXO1
TMPRSS2	https://www.genecards.org/cgi-bin/carddisp.pl?gene=TMPRSS2
MIR31	https://www.genecards.org/cgi-bin/carddisp.pl?gene=MIR31
HIF1A	https://www.genecards.org/cgi-bin/carddisp.pl?gene=HIF1A
MMP2	https://www.genecards.org/cgi-bin/carddisp.pl?gene=MMP2
MAPK1	https://www.genecards.org/cgi-bin/carddisp.pl?gene=MAPK1
IGF1	https://www.genecards.org/cgi-bin/carddisp.pl?gene=IGF1
MIR126	https://www.genecards.org/cgi-bin/carddisp.pl?gene=MIR126
MIR195	https://www.genecards.org/cgi-bin/carddisp.pl?gene=MIR195
POLE	https://www.genecards.org/cgi-bin/carddisp.pl?gene=POLE
MUTYH	https://www.genecards.org/cgi-bin/carddisp.pl?gene=MUTYH
MIR20A	https://www.genecards.org/cgi-bin/carddisp.pl?gene=MIR20A
MIR182	https://www.genecards.org/cgi-bin/carddisp.pl?gene=MIR182
CYP19A1	https://www.genecards.org/cgi-bin/carddisp.pl?gene=CYP19A1
IGF1R	https://www.genecards.org/cgi-bin/carddisp.pl?gene=IGF1R
MIR223	https://www.genecards.org/cgi-bin/carddisp.pl?gene=MIR223
MIR210	https://www.genecards.org/cgi-bin/carddisp.pl?gene=MIR210
MIR200B	https://www.genecards.org/cgi-bin/carddisp.pl?gene=MIR200B
ZFHX3	https://www.genecards.org/cgi-bin/carddisp.pl?gene=ZFHX3
MIR96	https://www.genecards.org/cgi-bin/carddisp.pl?gene=MIR96
MIR27A	https://www.genecards.org/cgi-bin/carddisp.pl?gene=MIR27A
RET	https://www.genecards.org/cgi-bin/carddisp.pl?gene=RET
MIR148A	https://www.genecards.org/cgi-bin/carddisp.pl?gene=MIR148A
MIR125A	https://www.genecards.org/cgi-bin/carddisp.pl?gene=MIR125A
CREBBP	https://www.genecards.org/cgi-bin/carddisp.pl?gene=CREBBP
STK11	https://www.genecards.org/cgi-bin/carddisp.pl?gene=STK11
TERC	https://www.genecards.org/cgi-bin/carddisp.pl?gene=TERC
MIR214	https://www.genecards.org/cgi-bin/carddisp.pl?gene=MIR214
NFKBIA	https://www.genecards.org/cgi-bin/carddisp.pl?gene=NFKBIA
MIR100	https://www.genecards.org/cgi-bin/carddisp.pl?gene=MIR100
EPCAM	https://www.genecards.org/cgi-bin/carddisp.pl?gene=EPCAM
PROS1	https://www.genecards.org/cgi-bin/carddisp.pl?gene=PROS1

BIRC5	https://www.genecards.org/cgi-bin/carddisp.pl?gene=BIRC5
MIR29A	https://www.genecards.org/cgi-bin/carddisp.pl?gene=MIR29A
PPARG	https://www.genecards.org/cgi-bin/carddisp.pl?gene=PPARG
MIR191	https://www.genecards.org/cgi-bin/carddisp.pl?gene=MIR191
MIR10B	https://www.genecards.org/cgi-bin/carddisp.pl?gene=MIR10B
CREB1	https://www.genecards.org/cgi-bin/carddisp.pl?gene=CREB1
MRE11	https://www.genecards.org/cgi-bin/carddisp.pl?gene=MRE11
CD44	https://www.genecards.org/cgi-bin/carddisp.pl?gene=CD44
PCA3	https://www.genecards.org/cgi-bin/carddisp.pl?gene=PCA3
KCNQ1OT1	https://www.genecards.org/cgi-bin/carddisp.pl?gene=KCNQ1OT1
TP63	https://www.genecards.org/cgi-bin/carddisp.pl?gene=TP63
ETV1	https://www.genecards.org/cgi-bin/carddisp.pl?gene=ETV1
MIR183	https://www.genecards.org/cgi-bin/carddisp.pl?gene=MIR183
TNFSF10	https://www.genecards.org/cgi-bin/carddisp.pl?gene=TNFSF10
MIR15A	https://www.genecards.org/cgi-bin/carddisp.pl?gene=MIR15A
NKX3-1	https://www.genecards.org/cgi-bin/carddisp.pl?gene=NKX3-1
JUN	https://www.genecards.org/cgi-bin/carddisp.pl?gene=JUN
MIR99A	https://www.genecards.org/cgi-bin/carddisp.pl?gene=MIR99A
POLD1	https://www.genecards.org/cgi-bin/carddisp.pl?gene=POLD1
MIR22	https://www.genecards.org/cgi-bin/carddisp.pl?gene=MIR22
MIR106B	https://www.genecards.org/cgi-bin/carddisp.pl?gene=MIR106B
MIR224	https://www.genecards.org/cgi-bin/carddisp.pl?gene=MIR224
MIR23B	https://www.genecards.org/cgi-bin/carddisp.pl?gene=MIR23B
C11orf65	https://www.genecards.org/cgi-bin/carddisp.pl?gene=C11orf65
MIR375	https://www.genecards.org/cgi-bin/carddisp.pl?gene=MIR375
IGFBP3	https://www.genecards.org/cgi-bin/carddisp.pl?gene=IGFBP3
MIR29B2	https://www.genecards.org/cgi-bin/carddisp.pl?gene=MIR29B2
MIRLET7B	https://www.genecards.org/cgi-bin/carddisp.pl?gene=MIRLET7B
MIR199B	https://www.genecards.org/cgi-bin/carddisp.pl?gene=MIR199B
MIR181A1	https://www.genecards.org/cgi-bin/carddisp.pl?gene=MIR181A1
BCL2L1	https://www.genecards.org/cgi-bin/carddisp.pl?gene=BCL2L1
MIRLET7D	https://www.genecards.org/cgi-bin/carddisp.pl?gene=MIRLET7D
RAD51	https://www.genecards.org/cgi-bin/carddisp.pl?gene=RAD51
MIR106A	https://www.genecards.org/cgi-bin/carddisp.pl?gene=MIR106A
MIRLET7C	https://www.genecards.org/cgi-bin/carddisp.pl?gene=MIRLET7C
MIAT	https://www.genecards.org/cgi-bin/carddisp.pl?gene=MIAT
MIR127	https://www.genecards.org/cgi-bin/carddisp.pl?gene=MIR127
TGFA	https://www.genecards.org/cgi-bin/carddisp.pl?gene=TGFA
MIR23A	https://www.genecards.org/cgi-bin/carddisp.pl?gene=MIR23A
CASP9	https://www.genecards.org/cgi-bin/carddisp.pl?gene=CASP9
MIR25	https://www.genecards.org/cgi-bin/carddisp.pl?gene=MIR25
FGF2	https://www.genecards.org/cgi-bin/carddisp.pl?gene=FGF2
CD36	https://www.genecards.org/cgi-bin/carddisp.pl?gene=CD36
KLK2	https://www.genecards.org/cgi-bin/carddisp.pl?gene=KLK2

MIR320A	https://www.genecards.org/cgi-bin/carddisp.pl?gene=MIR320A
MIRLET7G	https://www.genecards.org/cgi-bin/carddisp.pl?gene=MIRLET7G
IL1B	https://www.genecards.org/cgi-bin/carddisp.pl?gene=IL1B
MIR181B1	https://www.genecards.org/cgi-bin/carddisp.pl?gene=MIR181B1
PARP1	https://www.genecards.org/cgi-bin/carddisp.pl?gene=PARP1
MAPK3	https://www.genecards.org/cgi-bin/carddisp.pl?gene=MAPK3
POLK	https://www.genecards.org/cgi-bin/carddisp.pl?gene=POLK
MIR32	https://www.genecards.org/cgi-bin/carddisp.pl?gene=MIR32
DICER1	https://www.genecards.org/cgi-bin/carddisp.pl?gene=DICER1
E2F1	https://www.genecards.org/cgi-bin/carddisp.pl?gene=E2F1
MIR16-1	https://www.genecards.org/cgi-bin/carddisp.pl?gene=MIR16-1
CDK2	https://www.genecards.org/cgi-bin/carddisp.pl?gene=CDK2
IDH1	https://www.genecards.org/cgi-bin/carddisp.pl?gene=IDH1
CHGA	https://www.genecards.org/cgi-bin/carddisp.pl?gene=CHGA
SRD5A1	https://www.genecards.org/cgi-bin/carddisp.pl?gene=SRD5A1
MAK	https://www.genecards.org/cgi-bin/carddisp.pl?gene=MAK
ERBB3	https://www.genecards.org/cgi-bin/carddisp.pl?gene=ERBB3
MTHFR	https://www.genecards.org/cgi-bin/carddisp.pl?gene=MTHFR
MAPK8	https://www.genecards.org/cgi-bin/carddisp.pl?gene=MAPK8
PGR	https://www.genecards.org/cgi-bin/carddisp.pl?gene=PGR
MKI67	https://www.genecards.org/cgi-bin/carddisp.pl?gene=MKI67
GSTM1	https://www.genecards.org/cgi-bin/carddisp.pl?gene=GSTM1
MIR10A	https://www.genecards.org/cgi-bin/carddisp.pl?gene=MIR10A
BMP6	https://www.genecards.org/cgi-bin/carddisp.pl?gene=BMP6
CAV1	https://www.genecards.org/cgi-bin/carddisp.pl?gene=CAV1
IL10	https://www.genecards.org/cgi-bin/carddisp.pl?gene=IL10
CDKN2B-AS1	https://www.genecards.org/cgi-bin/carddisp.pl?gene=CDKN2B-AS1
PIK3R1	https://www.genecards.org/cgi-bin/carddisp.pl?gene=PIK3R1
MMP1	https://www.genecards.org/cgi-bin/carddisp.pl?gene=MMP1
CYCS	https://www.genecards.org/cgi-bin/carddisp.pl?gene=CYCS
MIR296	https://www.genecards.org/cgi-bin/carddisp.pl?gene=MIR296
EZH2	https://www.genecards.org/cgi-bin/carddisp.pl?gene=EZH2
MGMT	https://www.genecards.org/cgi-bin/carddisp.pl?gene=MGMT
PTK2	https://www.genecards.org/cgi-bin/carddisp.pl?gene=PTK2
GRP	https://www.genecards.org/cgi-bin/carddisp.pl?gene=GRP
SMARCA4	https://www.genecards.org/cgi-bin/carddisp.pl?gene=SMARCA4
DAB2IP	https://www.genecards.org/cgi-bin/carddisp.pl?gene=DAB2IP
TRPS1	https://www.genecards.org/cgi-bin/carddisp.pl?gene=TRPS1
SLC45A3	https://www.genecards.org/cgi-bin/carddisp.pl?gene=SLC45A3
SERPINA3	https://www.genecards.org/cgi-bin/carddisp.pl?gene=SERPINA3
HPC3	https://www.genecards.org/cgi-bin/carddisp.pl?gene=HPC3
MIR198	https://www.genecards.org/cgi-bin/carddisp.pl?gene=MIR198
ENO2	https://www.genecards.org/cgi-bin/carddisp.pl?gene=ENO2
PCAT1	https://www.genecards.org/cgi-bin/carddisp.pl?gene=PCAT1

CDK1	https://www.genecards.org/cgi-bin/carddisp.pl?gene=CDK1
MIR30B	https://www.genecards.org/cgi-bin/carddisp.pl?gene=MIR30B
FN1	https://www.genecards.org/cgi-bin/carddisp.pl?gene=FN1
HSP90AA1	https://www.genecards.org/cgi-bin/carddisp.pl?gene=HSP90AA1
PCAP	https://www.genecards.org/cgi-bin/carddisp.pl?gene=PCAP
PLAUR	https://www.genecards.org/cgi-bin/carddisp.pl?gene=PLAUR
AMACR	https://www.genecards.org/cgi-bin/carddisp.pl?gene=AMACR
GSK3B	https://www.genecards.org/cgi-bin/carddisp.pl?gene=GSK3B
MMP14	https://www.genecards.org/cgi-bin/carddisp.pl?gene=MMP14
HPC4	https://www.genecards.org/cgi-bin/carddisp.pl?gene=HPC4
HPC14	https://www.genecards.org/cgi-bin/carddisp.pl?gene=HPC14

Supplementary Table 4. PCa related gene results in OMIM

Cytogenetic location	Gene/Locus	Gene/LocusMIM number
1p22.1	BCAR3, SH2D3B, NSP2	604704
1q21.2	FALEC, FAL1	616092
1q32.1	BLACAT1, LINC00912	615480
1q42.2-q43	PCAP	602759
1q43-q44	SDCCAG8, CCCAP, SLSN7, BBS16	613524
1q43-q44	SDCCAG8, CCCAP, SLSN7, BBS16	613524
2p24.3	GACAT3, LINC01458	616132
2q12.2	C2orf40, ECRG4	611752
2q14-q21	LCO	165320
2q14.2	STEAP3, TSAP6, AHMIO2	609671
2q31.3	SCHLAP1, PCAT114, LINC00913	615568
2q32.2	DIRC1	606423
2q32.3	PCGEM1	605443
3p26	HPC5	609299
3p25-p22	OVCAS1	607893
3p23-p21	SCLC1	182280
3p22.2	DLEC1, DLC1	604050
3p21.31	TGM4	600585
3q22.1	ACPP	171790
3q27.1	EIF4G1, EIF4G, PARK18	600495
3q28	LNCR5	614210
4q13.3	PARM1, CIPAR1	617688
4q21.21	PCAT4, GDEP	609717
4q35.1	KMHN1	609488
5p15.33	LNCR3	612571
5p13.1	DAB2, DOC2	601236
5q12.1	PART1	604991
5q14.3	LUCAT1, SCAL1	618190
5q22.2	MCC	159350

6p24.3	CTAG3, CAGE1	608304
6p24.3	HULC	612210
6p22.3	CASC15, LINC00340	616610
6p21.33	LNCR4	612593
6q23-q25	LNCR1	608935
6q23.3	PBOV1, UROC28, UC28	605669
7p21.1	MACC1	612646
7p11-q21	HPC4	608658
7p11.2	VOPP1, ECOP, GASP	611915
7q21.12	STEAP4, STAMP2, TIARP	611098
7q21.13	STEAP	604415
7q21.13	STEAP2, STAMP1, IPCA1, PCANAP1	605094
7q22.1	GAEC1	612130
8p22	DLC1	604258
8p21.1	CLU, CLI, SGP2, TRPM2	185430
8q23	CRCS6	612231
8q24	CRCS2	611469
8q24	HPC10	611100
8q24.21	PCAT1	616043
8q24.21	PCAT2, PCA2, CARLO4	617678
8q24.21	PRNCR1, PCAT8	615452
8q24.21	CASC19, LINC01245, CARLO6	617703
8q24.21	CCAT1, CARLO5	617705
8q24.21	CASC21, LINC01244, CARLO2	617702
8q24.21	CASC8, LINC00860, CARLO1	617701
8q24.21	CASC11, LINC00990, CARLO7	617704
8q24.3	PSCA	602470
9p13.3	MSMP, PSMP	612191
9q21.2	PCA3, DD3	604845
9q33.1	DEC1	604767
10pter-q11	ST12, PAC1	601188
10p14	CRCS5	612230
10p11.23	MAP3K8, COT, EST, TPL2	191195
10q23.2	SNCG, BCSG1	602998
10q23.31	PTEN, MMAC1, GLM2, CWS1	601728
10q23.31	PTEN, MMAC1, GLM2, CWS1	601728
10q23.31	PTEN, MMAC1, GLM2, CWS1	601728
10q23.31	PTEN, MMAC1, GLM2, CWS1	601728
10q23.31	PTEN, MMAC1, GLM2, CWS1	601728
10q23.31	PTEN, MMAC1, GLM2, CWS1	601728
10q23.31	PTEN, MMAC1, GLM2, CWS1	601728
10q26.11	CASC2	608598
11p11.12	FOLH1, FOLH, PSM, PSMA	600934

11q13	HPC14	611958
11q13.2	BRMS1	606259
11q13.3	ORAOV1, TAOS1	607224
11q13.3	CTTN, EMS1	164765
11q14	GCRG224	610888
11q23	CRCS7	612232
11q23.1	COLCA1	615693
11q23.1	COLCA2	615694
11q24.2	HEPN1	611641
11q24.2	PATE	606861
12p12.1	CASC1, LAS1, PPP1R54	616906
12q24.31	CDK2AP1, DOC1	602198
13q13.1	AS3	605333
14q22.2	CRCS8	612589
15q12	HPC7	610321
15q23	PCAT29	616273
15q26.1	IQGAP1, SAR1	603379
16p13.13	BCAR4	613746
16q21	TEPP	610264
16q22.1	CRCS9	612590
16q23.1	BCAR1, CRKAS, CAS	602941
17p13.3	BCPR	113721
17p13.3	DPH1, DPH2L1, OVCA1, DEDSSH	603527
17p13.3	OVCA2	607896
17p13.3	HIC1	603825
17q12	HPC11	611955
17q21-q22	HPC9	610997
17q21.31	BRCA1, PSCP, BROVCA1, PNCA4, FANCS	113705
17q21.31	BRCA1, PSCP, BROVCA1, PNCA4, FANCS	113705
17q21.31	BRCA1, PSCP, BROVCA1, PNCA4, FANCS	113705
17q21.32	PRAC1	609819
17q23.2	PPM1D, WIP1, IDDGIP	605100
17q23.2	PPM1D, WIP1, IDDGIP	605100
18p11.22	GACAT2, MTCL1AS1	616131
18q11.2	PCAT18, LINC01092	617647
19p13.12	UCA1, LINC00178, CUDR	617500
19q	HPCQTL19	607592
19q13.2	PCAT19, LINC01190	618192
19q13.33	PTOV1, ACID2	610195
19q13.4	HPC15	611959

20p12.3	CRCS11	612592
20q11.21	BASE	607627
20q11.23	BLCAP, BC10	613110
20q13	HPC3	608656
20q13.12	NCOA3, AIB1, TNRC14	601937
20q13.31	PMEPA1, TMEPAI	606564
21q22.3	TFF1, BCEI	113710
22q11.21	HIC2, HRG22, KIAA1020	607712
22q12.3	HPC6	609558
Xp11.22	HPCX2	300704
Xq23	KKLC1, CXorf61	300625
Xq24	CT47B1, CT47A13	300790
Xq24	CT47A11, LOC255313	300592
Xq24	CT47A10	300789
Xq24	CT47A9	300788
Xq24	CT47A8	300787
Xq24	CT47A7	300786
Xq24	CT47A6	300785
Xq24	CT47A5	300784
Xq24	CT47A4	300783
Xq24	CT47A3	300782
Xq24	CT47A2	300781
Xq24	CT47A1	300780
Xq26.3	CT45A4, CT45.4	300795
Xq26.3	CT45A1, CT45	300648
Xq26.3	CT45A3, CT45.3	300794
Xq26.3	CT45A5, CT45.5	300796
Xq26.3	CT45A6, CT45.6	300797
Xq26.3	CT45A2, CT45.2	300793
Xq27-q28	HPCX1	300147
Xq27.1	LDOC1	300402
Xq28	CTAG1A, LAGE2A	300657
Xq28	CTAG1B, CTAG1	300156
Xq28	CTAG2, LAGE1, CAMEL	300396

Supplementary Table 5.

The data used in PPI network of HDW compound targets
PTGS1,CHRM3,CHRM1,SCN5A,CHRM5,PTGS2,CHRM4,RXRA,OPRD1,HRH1,CHRM2,ADRA2B,SLC6A3,ADRB2,CHRNA2,SLC6A4,DRD2,OPRM1,GABRA1,LTA4H,MAOB,NCOA2,PKIA,CASP3,PTGS2,PRSS1,GRIA2,GABRA1,PGR,NR3C2,PTGS2,PRSS1,GRIA2,PTGS1,PTGS2,PRSS1,GRIA2,HRH1,SLC6A2,ADRA1,A,CHRM2,ADRA1B,SLC6A3,ADRB2,ADRA1D,SLC6A4,OPRM1,GABRA1,NCOA2,PKIA,PGR,N

R3C2,NCOA2,ADH1C,RXRA,NCOA1,PTGS1,PTGS2,ADRA2A,SLC6A2,SLC6A3,ADRB2,AKR1B1,PLAU,PGR,PTGS1,CHRM3,KCNH2,CHRM1,SCN5A,PTGS2,HTR3A,RXRA,ADRA1B,ADRB2,ADRA1D,ADRA1A,CHRM2,ADRA1B,ADRB2,CHRNA2,SLC6A4,OPRM1,GABRA1,BCL2,BAX,CASP9,JUN,CASP3,CASP8,PRKCA,PON1,MAP2,PLAU,CTSB,RELA,STAT3,VEGFA,CCND1,BCL2,BCL2L1,FOS,CDKN1A,BAX,CASP9,MMP2,MMP9,TNFSF15,JUN,IL6,CASP3,TP63,MAPK8,PTGS2,NFKBIA,CASP8,FASN,MMP1,MMP3,MMP10,ICAM1,IL1B,CREB1,SELE,PTGER3,PTGS1,MCL1,PRKCG,ATF2,CSF2,PECAM1,MAPK8IP2,BIRC5,PTPN6,GAP43,DUOX2,NOS3,PTPN1,LITAF,CCND2,FASLG,CASP1,ENPP7,NFKBIA,POR,ODC1,CASP8,TOP1,RAF1,SOD1,PRKCA,MMP1,HIF1A,STAT1,RUNX1T1,ERBB2,PPARG,ACACA,HMOX1,CYP3A4,CYP1A2,CAV1,MYC,F3,GJA1,CYP1A1,ICAM1,IL1B,CCL2,SELE,VCAM1,PTGER3,CXCL8,PRKCB,BIRC5,DUOX2,NOS3,HSPB1,SULT1E1,MGAM,IL2,NR1I2,CYP1B1,CCNB1,PLAT,THBD,SERPINE1,COL1A1,IFNG,ALOX5,IL1A,MPO,TOP2A,NCF1,ABCG2,HAS2,GSTP1,NFE2L2,NQO1,PARP1,AHR,PSMD3,SLC2A4,COL3A1,CXCL11,CXCL2,DCAF5,NR1I3,CHEK2,INSR,CLDN4,PPARA,PPARD,HSF1,CRP,CXCL10,CHUK,SPP1,RUNX2,RASSF1,E2F1,E2F2,ACPP,CTSD,IGFBP3,IGF2,CD40LG,IRF1,ERBB3,PON1,DIO1,PCOLCE,NPEPPS,HK2,RASA1,GSTM1,GSTM2,PTGS1,PTGS2,GABRA1,TK1,PRSS1,GRIA2,ABCC2,TOP2.

Supplementary Table 6.

The data used in thePPI network of HWD compound targets against PCa
PTGS2，CASP3，PLAU，BCL2，BAX，CASP8，STAT3，VEGFA，CCND1，CDKN1A，MMP2，MMP9，IL6，TP63，NFKBIA，CREB1，BIRC5，RAF1，HIF1A，ERBB2，PPARG，CAV1，MYC，GSTP1，PARP1，AHR，CHEK2，RUNX2，ACPP，IGF2.

Supplementary Table 7.Detailed biological processes of HDW.

Biological Process	Adjusted p value	Node Size
DNA topological change	0.0041	2.83
Regulation of mitotic recombination	0.0043	2.83
Regulation of DNA recombination	0.0281	2.83
DNA strand elongation involved in DNA replication	0.0287	2.83
DNA strand elongation	0.0287	2.83
Negative regulation of TOR signaling cascade	0.0287	2.00
S-methylmethionine transport	0.0287	2.00
Positive regulation of transcription regulator activity	0.0287	2.00
Steroid acetylation	0.0287	2.00
Positive regulation of transcription factor activity	0.0287	2.00
Sterol acetylation	0.0287	2.00
Regulation of nucleobase, nucleoside, nucleotide, and nucleic acid metabolic process	0.0377	4.47
Regulation of nitrogen compound metabolic process	0.0377	4.47
Age-dependent response to reactive oxygen species	0.0451	2.00

Involved in chronological cell aging		
Chromatin organization	0.0473	3.46
Chromatin assembly or disassembly	0.0473	2.83
DNA conformation change	0.0473	2.83
Regulation of TOR signaling cascade	0.0473	2.00
Positive regulation of DNA binding	0.0473	2.00
Chromatin remodeling at centromere	0.0473	2.00
Regulation of DNA metabolic process	0.0483	2.83
